# Has COVID-19 changed how people think about the drivers of health? If so, does it matter?

**DOI:** 10.3389/frhs.2022.987226

**Published:** 2022-11-23

**Authors:** Christopher Nelson, Laurie T. Martin, Douglas Yeung, Delia Bugliari

**Affiliations:** ^1^RAND Corporation, Santa Monica, CA, United States; ^2^RAND Corporation, Washington, DC, United States

**Keywords:** health mindset, COVID-19, social determinants of health (SDOH), focusing events, pandemic

## Abstract

**Background:**

Could the COVID-19 pandemic prompt shifts in Americans' basic views on health mindset and policy solutions to health crises?

**Methods:**

A sample of 1,637 individuals rated the extent to which items (e.g., the role of environmental vs. individual factors) “may affect people's health and wellbeing,” both before (2018) and during the pandemic. In summer 2020 and fall 2021 they responded to questions about vaccination status and perceptions of COVID-19 related policies. We assessed changes in health mindset using repeated measures logistic regression, and used cross-sectional logistic regressions to assess whether variations in mindset explain COVID-19 related attitudes and behavior.

**Results:**

Between 2018 and 2021 respondents gave increasing weight to where people live and genetic factors and less weight to the role of individual health choices. Views on the importance of access to healthcare did not change appreciably. Those who reported that health care and place have a strong effect on health and wellbeing were significantly more likely to get vaccinated. Moreover, those who strongly believed that place is important were significantly less likely to agree that their local government went too far in restricting their freedom and that the local economy should have been left alone.

**Conclusion:**

Respondents were more likely in 2021 than in 2018 to recognize social determinants of health, and this is associated with a greater openness to pandemic-control measures. It remains to be seen, however, whether the changes in health mindset will persist over time and contribute to changes in policy and practice.

## Introduction

Previous research suggests that major historical events—including economic, political, and health crises—can lead to significant changes in basic beliefs and attitudes. For instance, a study 5 years after the Deepwater Horizon oil spill found that those affected were more likely to participate in political activities, make significant lifestyle changes, and have more concern for the environment (1). Pandemics, earthquakes, oil spills, and other disasters are examples of what political science and communication scholars have termed “focusing events,” (2) which may attract and channel attention to previously dormant issues (3, 4), create strong emotional reactions (e.g., fear, blame) (1), shape public opinion (5–7), impact trust in public institutions (8–10), officials and experts (10), and trigger physical and psychological health problems (11) and social unrest (12). In some cases, as in the Three Mile Island, Chernobyl, and Fukushima nuclear accidents, these events may also contribute to significant changes in policies and practices (5–7). As Bergstran and Mayer summarize, “it would appear that disasters do not just disrupt lives; they disrupt worldviews” (1) and this, under some circumstances, may contribute to policy and system change.

As such, some assert that the global health and economic crises brought by the coronavirus pandemic might create the opportunity for significant change in health mindset. Health mindset includes basic understandings of the factors that generate health, such as social determinants of health; perceptions around the relative roles of personal and environmental health influences; beliefs about equity; and expectations for society about who should be responsible for health and the forms of collective action needed to support sustained investments in it (e.g., clean water and air, walkable environments, green and blue spaces, robust public health systems) (13). Health mindset is broader than attitudes about specific policies, programs, actions, or policy actors. It may also influence individuals' support for specific policies (14) and investments related to public health and wellbeing, and their willingness to engage in collective action (15). Despite the evidence regarding factors influencing health outcomes, the American public has principally endorsed the disproportionate role of individual responsibility relative to other health influences, thus making some societal health policy measures more difficult to implement (16).

The COVID-19 pandemic has been the most disruptive health event in generations—possibly since the 1,918 pandemic and certainly since the influenza pandemic of 1957–58. It has had massive effects on U.S. health (nearly 1 million deaths as of May 2022), the economy [a 3.4% decline in Gross Domestic Product in 2020 (17) and over 22 million people out of work in March, 2020 (17)], and education [in February 2021, only one-third of students received full-time in-person instruction (18)]. It has introduced many people to an unfamiliar and evolving set of scientific concepts, how decisions get made under uncertainty, and the role of health risk and protective factors (e.g., masks, social distancing) that have all become central to political debates in the US and elsewhere. The question, therefore, is whether this pandemic might prompt mindset changes that could lead to significant changes in future health policy.

In this paper, we draw upon a unique longitudinal survey to assess the extent to which the COVID-19 pandemic has prompted shifts in Americans' basic views on health (i.e., “mindset”) and what this means for the potential success of policy solutions to health crises, such as broad mandates designed to protect the public's health. We seek to answer two specific questions. First, how, if at all, has health mindset changed during the pandemic, especially as it relates to views on the basic causes of good health? Second, to what extent are mindset changes likely to impact individual behavior (e.g., vaccine uptake) and perceptions of and support for policies and other collective actions designed to improve community health? Answering these questions might help public health responders better anticipate public reactions to disease control measures and inform efforts to improve public health systems as the nation recovers from the pandemic.

## Methods

### Data

In 2015 and 2018 RAND and the Robert Wood Johnson Foundation (RWJF) collaborated to develop and field the National Survey of Health Attitudes (NHSA), to help understand national perspectives on health-related attitudes, values and mindset (19, 20). In the context of COVID-19, RAND and RWJF drew from the NSHA to implement a second longitudinal survey, the COVID-19 and the Experiences of Populations at Greater Risk Survey (CEPGRS), to understand how these health views and values have been affected by the experience of the pandemic. Four waves of data were collected between the summer of 2020 and the fall of 2021 (21). For both surveys, respondents were recruited from two panels: the RAND American Life Panel (ALP) (22) and the KnowledgePanel (KP), administered by Ipsos (23). Both panels are nationally representative Internet panels whose members are recruited *via* probability-based sampling methods. Because the CEPRGS was only administered during the pandemic, we used a combined sample to examine changes in health mindset before and during the pandemic. Thus, our final analytic sample, which included participants who responded to both the NHSA and CEPGRS, ended up including more white and higher education (of all racial categories) respondents than the general national population. For cross-wave analysis, we calculated weights to align with national demographic distribution using the 2019 US Current Population Survey. Our weighting procedure is the same procedure used for other ALP surveys (24).

### Variables

This paper seeks to explain variation in respondent perception of four factors that may impact health: health care, the places they live, the choices they make, and how they were born (genetics/DNA). To measure health mindset, participants in both the NSHA and CEPGRS were asked to rate the extent to which items “may affect people's health and wellbeing” on a scale of 1 (no effect) to five (very strong effect). To reduce respondent burden in the CEPGRS survey instrument, several items from the NSHA were combined. For example, “smoking” and “health behaviors excluding smoking” were combined into “choices they make about their diet, exercise, smoking, etc.” In creating a common dataset that utilized questions from both surveys, we had to combine responses on the NSHA to match the structure of the CEPGRS. We did this by taking the average of relevant NSHA items corresponding to each of the four health mindset dimensions to generate a single rating for 2018. Individuals were considered to agree that an item affected people's health if they rated it a four (strong effect) or higher.

Individual behavior and perceptions of policy were characterized using data from Wave 4 of the CEPGRS, which included several questions related to COVID-19. To determine vaccination status, we used responses to the question “Have you completed your vaccinations, meaning two shots (Pfizer, Moderna), or one shot (Johnson and Johnson)?” To examine policy, we sought items that were timely and salient during the pandemic, including contentious debates over the relative weight given to public health vs. the economy. Specifically, respondents were asked to rate the extent to which they agreed with the statements, “During the course of the pandemic, my local government has gone too far in restricting my freedom to move about” and “During the course of the pandemic, the economy in my area should have been kept open.”

### Analysis

We used repeated measures logistic regression to detect change in individuals' health mindset across three survey years: 2018 (NSHA), 2020 (Wave 1 of CEPGRS), and 2021 (Wave 4 of CEPGRS).^1^ For the purposes of our analyses, we dichotomized responses into agree (strongly agree and somewhat agree) and do not agree (neither agree nor disagree, somewhat disagree, strongly disagree). To address concerns that any detected trends may be driven by our selection of a cut point for dichotomizing the, we conducted a sensitivity analysis, dichotomizing responses instead as little/no effect compared to at least some effect (some/strong/very strong). While slightly attenuated, the directionality and significance of observed trends did not change.

To examine the relationship between health mindset, vaccine status, and perceptions of COVID-related policies, we used Wave 4 data to conduct logistic regressions controlling for respondent race/ethnicity, income, age, gender, education, and urban/rural residence. These model specification decisions were informed by earlier work that analyzed 2018 data from the same survey that found differences among racial/ethnic groups in beliefs about social determinants of health (25). We subsequently added urban/rural residence to the models as a rough proxy for local-level differences in factors, but did not observe any change in results. We included all mindset variables after confirming that multicollinearity among the variables was not high enough to substantially impact the estimates and confirming that estimating separate regressions for each yielded similar results.

## Results

### Respondent characteristics

Table 1 summarizes the characteristics of respondents in the sample. The final analytic sample is more white (73% non-Hispanic white), older (86% at least age 45) and more highly educated (54% college graduates) than the U.S. population (26).

**Table 1:** Summary statistics of analytic sample (*n* = 1,637).

**Variable**	***N* (%)**
**Race/Ethnicity**	
Non-Hispanic (NH) white	1,198 (73%)
NH Black	132 (8%)
Hispanic	208 (13%)
NH Asian/Pacific Islander	44 (3%)
NH other	55 (3%)
**Household Income**	
Less than $10k	61 (4%)
$10k−24,999	156 (10%)
$25k−49,999	382 (23%)
$50k−74,999	350 (21%)
$75k−99,999	210 (13%)
$100k+	475 (29%)
**Age**	
25–44	227 (14%)
45–64	667 (41%)
65+	743 (45%)
***Gender***	
Male	718 (44%)
Female	919 (56%)
***Education***	
Some high school or less	41 (3%)
High school degree	180 (11%)
Some college	537 (33%)
Bachelor's degree or higher	879 (54%)
***Urbanicity***	
Small to midsize city or large city, 50K+ population	1,273 (78%)
Rural or small town, population under 50K	362 (22%)

### Changes in mindset

**Figure 1:**
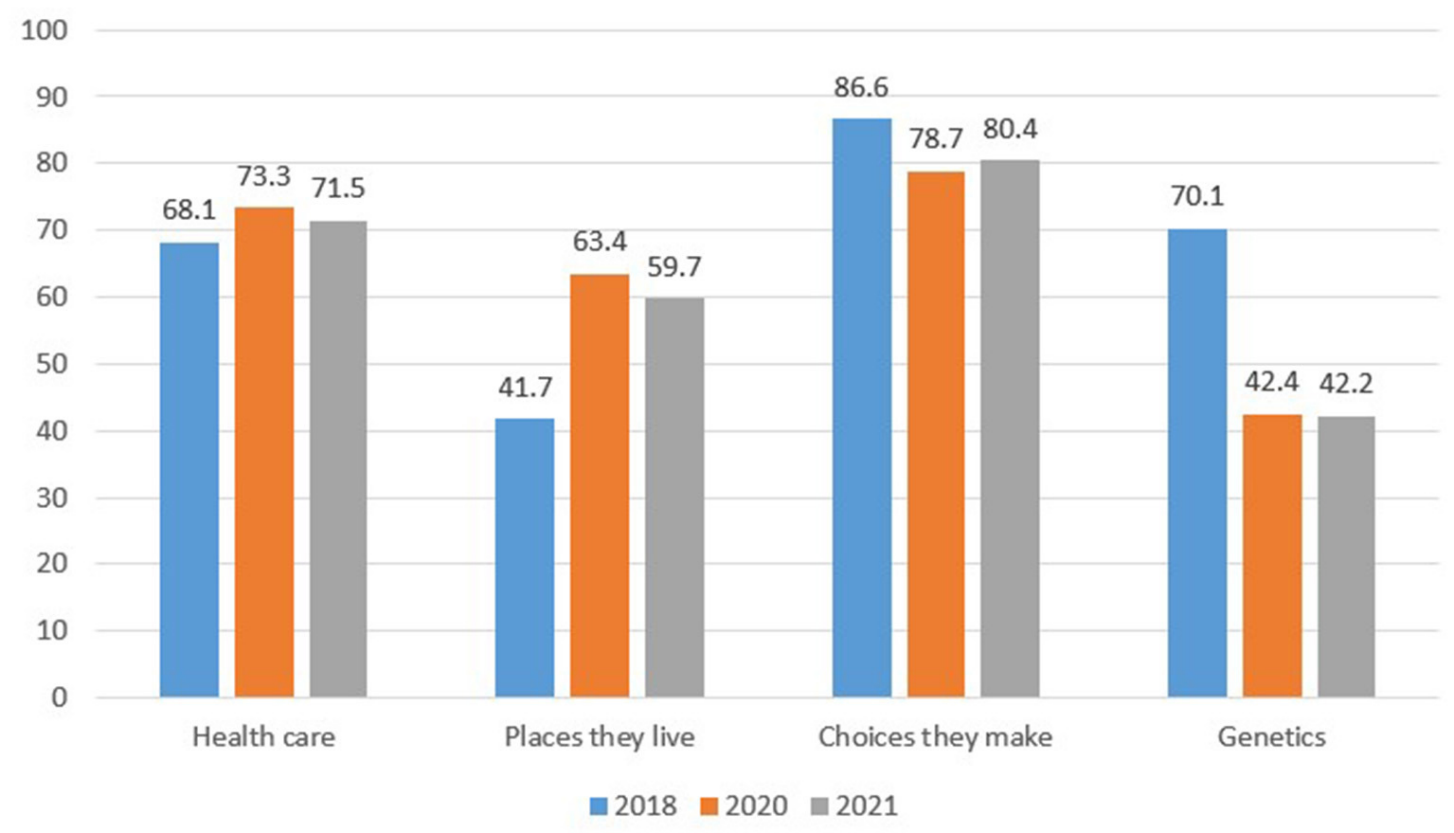
Mindset change over time—Percent of respondents rating mindset items as having “strong/very strong effect” (weighted). Source: Authors.

Our first research question was how, if at all, has health mindset changed during the pandemic, especially as it relates to views on the basic causes of good health? Figure 1 illustrates how respondents' beliefs about what affects health and wellbeing changed over the course of the pandemic. Respondents' beliefs about the role of health care remained largely unchanged (*p* = 0.25). There was a slight decrease in the belief that people's choices impact their health (*p* = 0.003), but an increase in the belief that where people live had an impact on health and wellbeing (*p* < 0.001). There was a decrease in the belief that how people were born (*p* < 0.001) impacts health. We note, however, that the wording of the “how born” item changed between the NHSA and the CEPGRS (i.e., previously it had been phrased as “genetic makeup inherited from parents”). Thus, some of the observed change from 2018 may reflect the change in the language.

### Health mindset and vaccination status

The second research question was to what extent are mindset changes likely to impact individual behavior (e.g., vaccine uptake) and perceptions of and support for policies and other collective actions designed to improve community health? Using cross-sectional data from Wave 4, we examined the association between health beliefs/mindset and vaccination status. Here, we grouped each belief/mindset into three categories: strong or very strong effect, some effect, and little effect or no effect. After controlling for race/ethnicity, income, age, gender, education, and rural/urban residence, those who reported that *health care* and *place* have a strong effect on health and wellbeing were both significantly more likely to get vaccinated (Table 2). Those who strongly believe that health is driven by *choices* were about half as likely to be vaccinated, although this difference was not statistically significant.

**Table 2:** How mindset matters for individual behavior: Logistic regression results predicting receipt of COVID-19 vaccine, controlling for demographic variables.^a^

**Health mindset (i.e., beliefs about drivers of health)**	**Odds ratio (95% Confidence Interval)**
Health care they get, no effect	–
Health care they get, some effect	1.93 (0.95–3.90)
Health care they get, strong effect	4.56 (2.27–9.15)^**^
Place they live, no effect	–
Place they live, some effect	1.13 (0.68–1.89)
Place they live, strong effect	2.02 (1.20–3.39)^**^
Choices they make, no effect	–
Choices they make, some effect	0.95 (0.41–2.23)
Choices they make, strong effect	0.49 (0.22–1.09)
How born, no effect	–
How born, some effect	0.64 (0.41–1.00)
How born, strong effect	0.74 (0.47–1.16)

a Models control for race/ethnicity, family income, age, gender, education, and urban/rural. *p < 0.05 and

** *p* < 0.01.

### Health mindset and perception of COVID-19 policies

**Figure 2:**
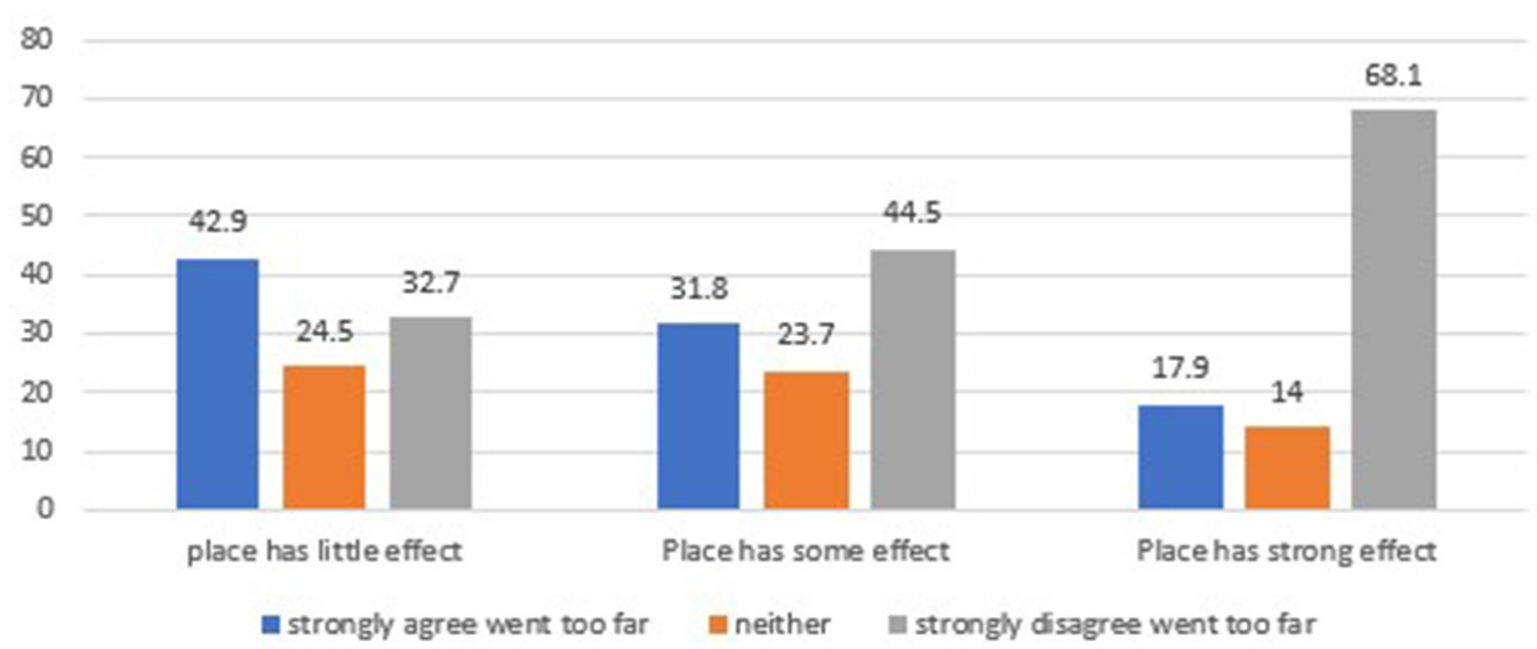
How mindset matters for policy—Relationship between belief about the importance of place and the belief that “During the course of the pandemic, my local government (city or county) has gone too far in restricting my freedom to move about.” Source: Authors.

Using cross-sectional data from Wave 4, we also examined whether one's health mindset was associated with perceptions of COVID-19 related policies. In a model that included each health belief (referenced above) and controlled for race/ethnicity, family income, age, gender, education, and rural/urban residence, those who strongly believed that *place* is important were significantly less likely to agree that their local government went too far in restricting their freedom (OR = 0.36; 95% CI 0.23–0.56). No other associations between health mindset and belief that local government went too far in restrictions were statistically significant. Figure 2 provides an unweighted breakdown of beliefs about place and perceptions that local government went too far. Of those who believe place has a strong effect, about 18 percent felt government went too far, compared to about 43 percent among those who believe place has little to no effect on health.

**Figure 3:**
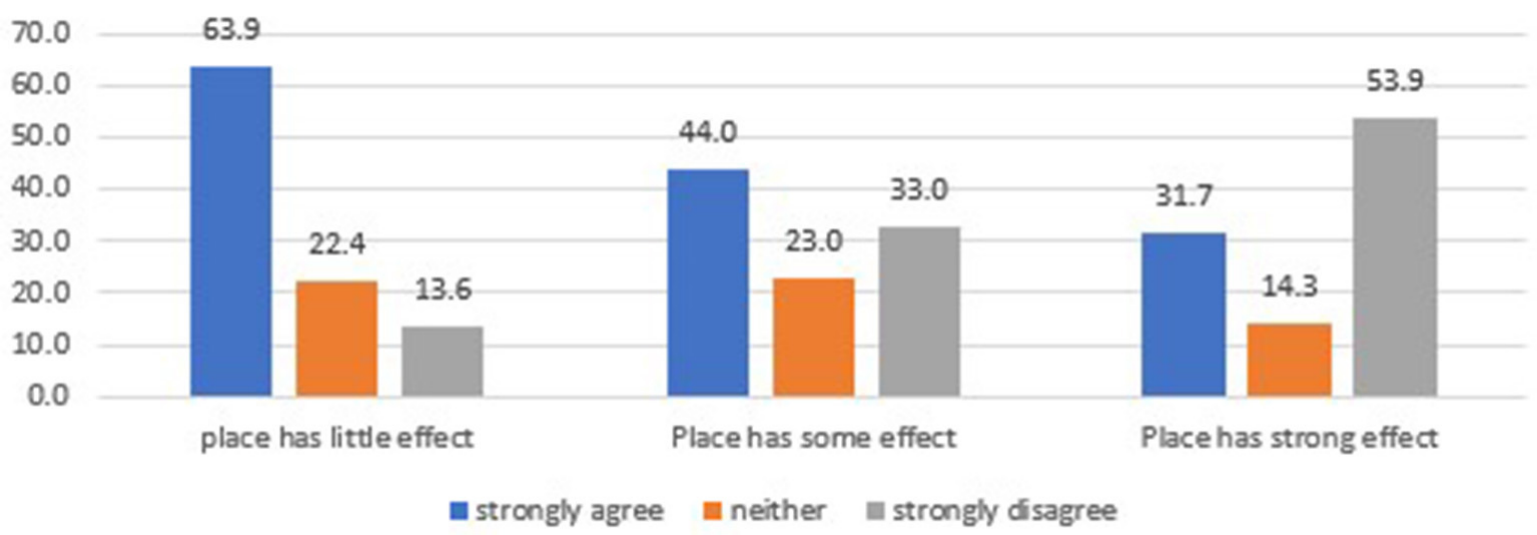
How mindset matters for policy—Relationship between one's belief about the importance of place for health and wellbeing and the belief that “During the course of the pandemic, the economy in my area (city or county) should have been kept open.” Source: Authors.

We also examined whether one's health mindset was associated with the belief that the economy in their area should have been kept open. Individuals were significantly less likely to believe that their local economy should have been left open if they reported that *place* has some effect (OR = 0.40, 95% CI 0.25–0.63) or a strong effect (OR = 0.28, 95% CI 0.18–0.44) on health. Over half of those who believe place has a strong effect on health strongly disagreed with the statement that the local economy should have been kept open, while well over half of those who believe place has little effect felt the local economy should have been kept open (Figure 3).

## Discussion

The history of public health suggests that it can be difficult to produce sustained improvement in health outcomes without attention to the complex interplay of individual and community influences. As Frieden (27) observed, “it makes little sense to expect individuals to behave differently than their peers; it is more appropriate to seek a general change in behavioral norms and in the circumstances which facilitate their adoption” [cited in (27)]. It is increasingly clear, moreover, that mindset is among these upstream circumstances (13, 28). In this research, we sought to assess whether basic beliefs about the drivers of health have changed during the COVID-19 pandemic. Our analysis suggests that between 2018 and 2021 respondents gave increasing weight to place and genetic factors as drivers of health and less weight to individual choices. Views on the importance of access to healthcare, by contrast, did not change appreciably.

Probing the specific mechanisms behind these changes is beyond the scope of this paper. However, we note that a common thread in this pattern of findings is that respondents were more likely in 2021 than in 2018 to endorse statements that emphasize an appreciation for community or environmental factors influencing health, which could include an increased understanding of the role of social determinants of health (29, 30). While some people—particularly those with more resources and education—can change their location of residence (31, 32), such opportunities are not available to the broader population. Overall, this increased appreciation of sense of place seems consistent with the realities of widespread prevalence of a potentially infectious disease, in which an individual's health might be compromised by contact with others whose choices are outside their direct control.

Our analysis also shows some of the ways in which mindset might impact individual behavior. While beliefs about the importance of access to healthcare did not change from 2018 to 2021, our analysis does suggest that those who rate healthcare as an important driver of health are more likely to report getting vaccinated. This is not surprising, as it is likely that respondents view vaccination as part of “health care,” even though public health professionals may regard it as a “population health” measure. Similarly, those who rate place as an important driver of health were also more likely to report getting vaccinated, which is consistent with a greater appreciation for community-level drivers of pandemic risk, and affirmed by research that demonstrates how COVID-19 risk varies considerably with location (33, 34).

If the findings do, in fact, indicate an increased appreciation of social determinants of health, a logical question is whether this could translate into support for policies, actions by business, or other forms of collective action to address common threats to health and long-standing weaknesses (35, 36) and underinvestment (37) in the U.S. public health system. Indeed, studies of health social movements (38, 39) have underscored the importance of changes in mindset—along with research, civic engagement, and policy ideas (40)—as important drivers of systemic change.

Indeed, we do find that beliefs about the role of place predicted respondents' evaluations of whether efforts to stem the pandemic went too far in restricting freedom of movement and economic activity. Here, the relationship was stronger for the belief that the economy should have been kept open than for restrictions on freedom of movement. One possible explanation for this difference is that economic effects were more salient to respondents given its impacts on their financial wellbeing.

However, there are reasons to doubt that such changes in mindset could trigger broad efforts toward system change. The consequences of pandemics play out over a long period of time and the effects are often spread unevenly, meaning that the real or perceived risk to one's health can be quite variable. Perhaps more importantly, with the exception of those directly affected, the worst suffering is largely out of sight, confined to hospital intensive care units. The fact that (in spite of this) we observe discernible changes in such fundamental beliefs about health is notable, especially given the deep politicization of the pandemic (41, 42). That said, there is reason to suspect such changes may attenuate over time, as was the case with attitudes about work and war, patriotism, and risk perceptions after 9/11, where research found that many of the shifts had dissipated or reverted within months or a year (11).

There are, of course, important limitations to our analysis. First, the sample used was limited to those who responded to the NHSA in 2018 and both the first and final waves of the CEPGRS (2020 and 2021, respectively). While the CEPGRS oversampled vulnerable populations for its main research aim, our interest in assessing changes over time necessitated limiting our sample to those for whom data were available at all three time points. As such, the final analytic sample is whiter (73% non-Hispanic white), older (86% at least age 45) and more highly educated (54% college graduates) than the U.S. population. Second, we note that while our survey data allow us to assess the link between health mindset and respondents' assessment of past policy actions in their communities, they do not allow us to assess support for future policies. Third, there may be important community-level factors (e.g., vaccine availability, ambient culture) that go beyond the individual-level attributes we measure and could impact respondents' mindset and behaviors. Given the design of the study, our ability to link community factors to individual respondents was limited, but we sought to address this limitation by including a measure of rural/urban residence, which may serve as a rough proxy for such community level factors. Finally, we collapsed the 5-point scales into two- and three-level variables. While this resulted in some loss of information, such recodes helped to address low cell sizes in the tails of the distribution and facilitated interpretation and presentation of findings.

## Conclusion

The COVID-19 pandemic appears to have triggered discernible shifts in health mindset, particularly on the importance of social determinants of health. Belief in the importance of these factors also seems to be predictive of individual protective behavior (e.g., vaccination) and support for policy and other restrictions on the economy and freedom of movement. Previous literature on social movements suggests that changes in mindset can be an important ingredient in significant policy and system change. But it remains to be seen whether the changes in health mindset will persist over time and result in practical changes. In the meantime, it may be wise to add health mindset to the set of health beliefs and behaviors (43) monitored by those planning for and responding to pandemics, both now and in the future.

## Data availability statement

The raw data supporting the conclusions of this article will be made available by the authors, without undue reservation, and will eventually be made available in ICPSR.

## Ethics statement

The studies involving human participants were reviewed and approved by the RAND Human Subjects Protection Committee (ref: 2014-0336). The patients/participants provided their written informed consent to participate in this study before taking part.

## Author contributions

CN, LM, and DY: conception of work. LM, DY, CN, and DB: design of the work, interpretation of data, drafted the work, and substantively revised it. DB and LM: data acquisition and analysis. All authors read and approved the final manuscript.

## Funding

Funding was provided by Robert Wood Johnson Foundation, Contract #74430.

## Conflict of interest

Authors CN, LM, DY, and DB are employed by RAND Corporation, a nonprofit, nonpartisan research organization. The authors declare that the research was conducted in the absence of any commercial or financial relationships that could be construed as a potential conflict of interest.

## Publisher's note

All claims expressed in this article are solely those of the authors and do not necessarily represent those of their affiliated organizations, or those of the publisher, the editors and the reviewers. Any product that may be evaluated in this article, or claim that may be made by its manufacturer, is not guaranteed or endorsed by the publisher.

## Footnotes

^1^The “svyset” capability within statistical software Stata was used to correct standard errors for survey design by specification of sampling weights and Taylor variance estimation.
