# Supplementary material for: Has COVID-19 changed how people think about the drivers of health? If so, does it matter?
Source: Front Health Serv. 2022 Nov 23;2:987226. doi: 10.3389/frhs.2022.987226 (PMC10012659; doi:10.3389/frhs.2022.987226)
Supplement: Supplementary file 1 [file Table_1.pdf]

Appendix: Full Regression Output

Full results of logistic regression predicting receipt of COVID-19 vaccination

Model covariates	Coefficient (SE)	Odds Ratio (95% Confidence Interval)
**Health care they get**		
no effect	reference category	
some effect	0.648*	1.912*
	(0.361)	(0.943 - 3.880)
strong effect	1.516***	4.552***
	(0.356)	(2.266 - 9.145)
**Place they live**		
no effect	reference category	
some effect	0.130	1.138
	(0.261)	(0.682 - 1.900)
strong effect	0.708***	2.029***
	(0.266)	(1.206 - 3.415)
**Choices they make**		
no effect	reference category	
some effect	-0.0517	0.950
	(0.435)	(0.405 - 2.226)
strong effect	-0.718*	0.488*
	(0.408)	(0.219 - 1.084)
**How they were born**		
no effect	reference category	
some effect	-0.447*	0.639*
	(0.229)	(0.408 - 1.001)
strong effect	-0.296	0.743
	(0.232)	(0.472 - 1.170)
**Race/Ethnicity**		
white	reference category	
Black (non-Hispanic)	0.148	1.159
	(0.279)	(0.670 - 2.005)
Hispanic	0.0605	1.062
	(0.218)	(0.693 - 1.628)
Asian/PI (non-Hispanic)	0.741	2.099
	(0.647)	(0.590 - 7.464)
Other (non-Hispanic)	-0.354	0.702
	(0.355)	(0.350 - 1.408)

**Family income level**		
<$10,000	reference category	
$10k-24,999	0.176	1.192
	(0.375)	(0.572 - 2.486)
$25k-49,999	-0.00551	0.995
	(0.347)	(0.504 - 1.961)
$50k-74,999	0.242	1.274
	(0.357)	(0.632 - 2.567)
$75k-99,999	0.285	1.330
	(0.391)	(0.618 - 2.862)
$100k+	0.901**	2.462**
	(0.374)	(1.182 - 5.128)
**Age**		
<45	reference category	
45-64	0.364*	1.439*
	(0.207)	(0.959 - 2.159)
65+	1.010***	2.745***
	(0.223)	(1.772 - 4.254)
**Sex**		
male	reference category	
female	-0.0549	0.947
	(0.152)	(0.703 - 1.275)
**Highest level of education**		
<HS	reference category	
HS degree	0.321	1.378
	(0.416)	(0.610 - 3.112)
some college	0.478	1.612
	(0.399)	(0.738 - 3.521)
college degree	1.070***	2.915***
	(0.412)	(1.299 - 6.539)
**Urbanicity**		
urban	reference category	
rural	-0.510***	0.601***
	(0.166)	(0.433 - 0.832)
Constant	-0.623	0.537
	(0.588)	(0.170 - 1.698)
Observations	1,623	

Standard errors in parentheses
*** p<0.01, ** p<0.05, * p<0.1

Full results of logistic regression predicting the belief that the economy should have been kept open during the COVID-19 pandemic

Model covariates	Coefficient (SE)	Odds Ratio (95% Confidence Interval)
**Health care they get**		
no effect	reference category	
some effect	0.672**	1.958**
	(0.342)	(1.002 - 3.824)
strong effect	-0.245	0.783
	(0.330)	(0.410 - 1.494)
**Place they live**		
no effect	reference category	
some effect	-0.914***	0.401***
	(0.230)	(0.255 - 0.630)
strong effect	-1.248***	0.287***
	(0.226)	(0.184 - 0.447)
**Choices they make**		
no effect	reference category	
some effect	0.0540	1.056
	(0.379)	(0.503 - 2.217)
strong effect	0.497	1.643
	(0.359)	(0.814 - 3.318)
**How they were born**		
no effect	reference category	
some effect	0.0564	1.058
	(0.170)	(0.758 - 1.477)
strong effect	-0.0886	0.915
	(0.169)	(0.657 - 1.276)
**Race/Ethnicity**		
white	reference category	
Black (non-Hispanic)	-1.118***	0.327***
	(0.248)	(0.201 - 0.531)
Hispanic	-0.746***	0.474***
	(0.187)	(0.329 - 0.684)
Asian/PI (non-Hispanic)	-0.376	0.686
	(0.362)	(0.337 - 1.396)
Other (non-Hispanic)	-0.427	0.653

	(0.316)	(0.351 - 1.213)
**Family income level**		
<$10,000	reference category	
$10k-24,999	0.414	1.512
	(0.377)	(0.722 - 3.168)
$25k-49,999	0.925***	2.521***
	(0.352)	(1.264 - 5.028)
$50k-74,999	1.079***	2.943***
	(0.356)	(1.464 - 5.917)
$75k-99,999	1.039***	2.827***
	(0.372)	(1.364 - 5.858)
$100k+	0.921***	2.513***
	(0.357)	(1.248 - 5.058)
**Age**		
<45	reference category	
45-64	0.259	1.295
	(0.178)	(0.913 - 1.837)
65+	-0.105	0.901
	(0.183)	(0.629 - 1.289)
**Sex**		
male	reference category	
female	-0.353***	0.703***
	(0.112)	(0.565 - 0.875)
**Highest level of education**		
<HS	reference category	
HS degree	-0.551	0.577
	(0.410)	(0.258 - 1.288)
some college	-0.332	0.718
	(0.390)	(0.334 - 1.542)
college degree	-0.798**	0.450**
	(0.396)	(0.207 - 0.978)
**Urbanicity**		
urban	reference category	
rural	0.382***	1.465***
	(0.130)	(1.135 - 1.891)
Constant	-0.0456	0.955
	(0.566)	(0.315 - 2.895)

Observations	1,632

Standard errors in parentheses
*** p<0.01, ** p<0.05, * p<0.1

Full results of logistic regression predicting the belief that local government went too far in restricting freedoms during COVID-19 pandemic

Model covariates	Coefficient (SE)	Odds Ratio (95% Confidence Interval)
**Health care they get**		
no effect	reference category	
some effect	0.205	1.227
	(0.334)	(0.637 - 2.362)
strong effect	-0.578*	0.561*
	(0.328)	(0.295 - 1.067)
**Place they live**		
no effect	reference category	
some effect	-0.394*	0.674*
	(0.222)	(0.436 - 1.042)
strong effect	-0.990***	0.372***
	(0.222)	(0.241 - 0.574)
**Choices they make**		
no effect	reference category	
some effect	-0.0728	0.930
	(0.380)	(0.441 - 1.960)
strong effect	0.304	1.355
	(0.358)	(0.672 - 2.731)
**How they were born**		
no effect	reference category	
some effect	0.248	1.281
	(0.186)	(0.891 - 1.843)
strong effect	0.0439	1.045
	(0.188)	(0.723 - 1.509)
**Race/Ethnicity**		
white	reference category	
Black (non-Hispanic)	-0.336	0.715
	(0.251)	(0.437 - 1.168)
Hispanic	-0.318	0.727
	(0.201)	(0.491 - 1.078)
Asian/PI (non-Hispanic)	0.163	1.177
	(0.379)	(0.560 - 2.475)
Other (non-Hispanic)	-0.179	0.836
	(0.340)	(0.429 - 1.630)
**Family income level**		

<$10,000	reference category	
$10k-24,999	0.453	1.572
	(0.397)	(0.722 - 3.426)
$25k-49,999	0.740**	2.095**
	(0.373)	(1.008 - 4.353)
$50k-74,999	0.620	1.859
	(0.379)	(0.884 - 3.911)
$75k-99,999	0.773*	2.165*
	(0.398)	(0.993 - 4.721)
$100k+	0.597	1.817
	(0.380)	(0.863 - 3.826)
**Age**		
<45	reference category	
45-64	0.227	1.255
	(0.193)	(0.860 - 1.831)
65+	-0.257	0.773
	(0.202)	(0.520 - 1.149)
**Sex**		
male	reference category	
female	-0.275**	0.760**
	(0.125)	(0.595 - 0.971)
**Highest level of education**		
<HS	reference category	
HS degree	-0.642	0.526
	(0.411)	(0.235 - 1.177)
some college	-0.609	0.544
	(0.391)	(0.253 - 1.170)
college degree	-0.889**	0.411**
	(0.398)	(0.189 - 0.897)
**Urbanicity**		
urban	reference category	
rural	0.210	1.233
	(0.145)	(0.929 - 1.638)
Constant	-0.275	0.759
	(0.567)	(0.250 - 2.309)
Observations	1,631	

Standard errors in parentheses
*** p<0.01, ** p<0.05, * p<0.1
